# Late Presentation of β-Thalassemia Major Patient With Left Hemiparesis: A Case Report

**DOI:** 10.7759/cureus.52280

**Published:** 2024-01-15

**Authors:** Ashwaq Hussain, Ajay Singh, Sanjiya Arora, Varnika Gupta, Priya R Mallimala

**Affiliations:** 1 Hematology, Thalassemia and Sickle Cell Society, Kurnool, IND; 2 Internal Medicine, Sri Ram Murti Smarak Institute of Medical Sciences, Bareilly, IND; 3 Internal Medicine, Rohilkhand Medical College and Hospital, Bareilly, IND; 4 Internal Medicine, Lala Lajpat Rai Memorial Medical College, Meerut, IND; 5 Internal Medicine, Kurnool Medical College, Kurnool, IND

**Keywords:** iron overload, blood transfusion, cerebral atrophy, hemiparesis, beta-thalassemia

## Abstract

Thalassemia is a hereditary autosomal recessive disorder that is distinguished by a diminished rate of hemoglobin (Hb) synthesis arising from an anomaly in the synthesis of α or β globin chains. Classical symptoms of β-thalassemia are frequently observed in patients who present late for blood transfusion (BT), which is typical among South Asian countries in light of their limited resources. This case report is an uncommon instance of a typical occurrence that has been infrequently reported in the South Asian region. The reporting of this case will assist healthcare workers in managing cases appropriately. We present a 12-year-old female child diagnosed with β-thalassemia major with a late presentation than usual accompanied by an unusual finding of left hemiparesis at a young age of five years. The patient had been lost to follow-up, presented with easy fatiguability, poor weight gain, and growth restriction, all of which are classic symptoms of β-thalassemia. The patient was treated with a BT and continued to be monitored for transfusion and iron overload management.

## Introduction

β-thalassemia, a hereditary blood disorder, stands as one of the most intriguing and challenging conditions in the realm of hematology affecting approximately 1 in 100000 births globally [1]. In certain regions, especially those with higher rates of consanguinity, the prevalence of β-thalassemia is disproportionately elevated. For instance, Mediterranean countries, parts of South Asia, and the Middle East experience a higher incidence due to a higher frequency of carriers within these populations. This regional concentration poses challenges in diagnosis and management, as healthcare infrastructure and resources may vary, affecting the accessibility of specialized care and genetic testing. Socio-economic disparities contribute significantly to challenges in β-thalassemia diagnosis and management. Affordability and accessibility to advanced diagnostic tools, genetic counseling, and specialized treatments vary widely. Disparities in healthcare infrastructure and resources between developed and developing regions further exacerbate challenges in β-thalassemia diagnosis and management. This disparity underscores the need for global health initiatives to address the unequal burden of β-thalassemia.

Diagnosing β-thalassemia involves clinical evaluation, complete blood count (CBC) analysis, hemoglobin (Hb) electrophoresis, and genetic testing to identify specific mutations. The severity ranges from mild carriers to individuals needing regular blood transfusions. Management requires a multidisciplinary approach, including transfusions to alleviate anemia and chelation therapy to remove excess iron from transfusions. The uniqueness of the disease lies in its global distribution and genetic diversity, posing challenges for a universal cure. β-thalassemia disrupts the synthesis of Hb, leading to anemia and various clinical manifestations. The pathology involves mutations in the β-globin chains of Hb, resulting in an imbalance of alpha and beta chains and causing premature destruction of red blood cells (RBCs) [2]. These diverse genetic mutations influence the severity of the disease, response to treatment, and the complexity of clinical management, emphasizing the importance of individualized care strategies based on the specific genetic profile of each patient. Nevertheless, gene therapy and stem cell transplantation offer potential treatments and hope for a permanent cure [3].

β-thalassemia's complexity and geographic distribution make it a captivating yet challenging blood disorder, necessitating further research and innovative approaches to improve patient outcomes and enhance their quality of life. Herein, we expound upon an exceptional instance of a 12-year-old female who has been diagnosed with β-thalassemia major, exhibiting tardy presentation compared to the norm, in addition to an atypical discovery of left hemiparesis at the age of five years.

## Case presentation

**Figure 1:**
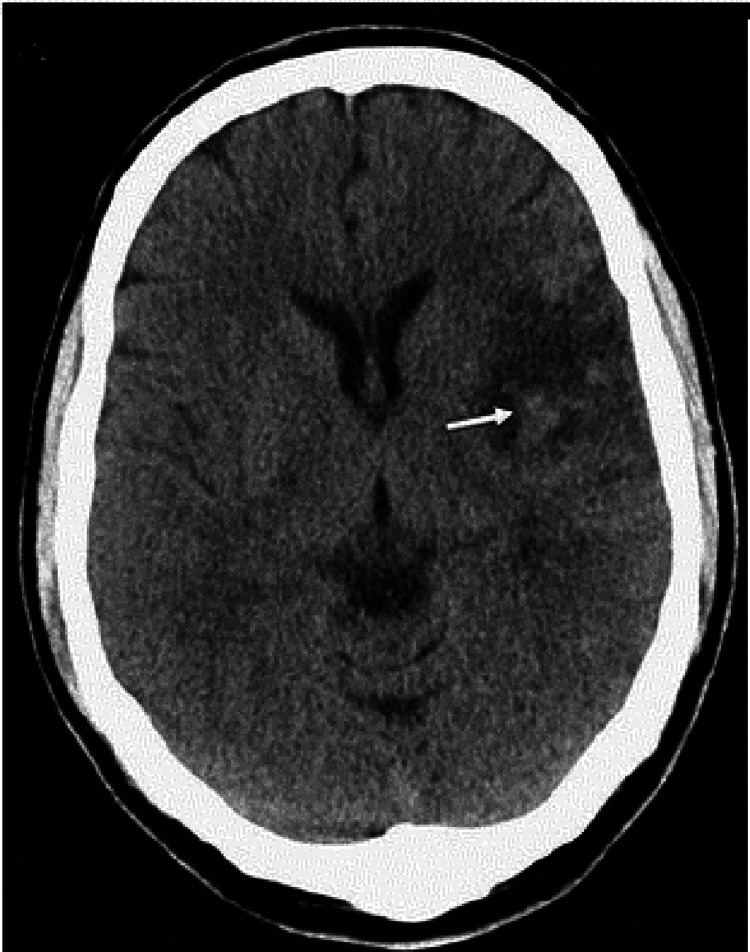
Hypodensities seen on right frontal, temporal, and parietal regions (arrow) suggestive of infarcts.

A 12-year-old female child first born of second-degree consanguineous marriage was brought to the outpatient department with symptoms of easy fatigability, not gaining weight, and growth restriction from the past year, with a previous history of five blood transfusions (BT). The first BT was at the age of five years, and the last BT was one month prior to presenting at our hospital. The patient's pre-transfusion hemoglobin (Hb) was 8 gm, had a history of left hemiparesis which was preceded by high-grade fever at the age of five years that required hospital admission (Figure 1). Following the stroke, the patient experienced difficulties in performing fine motor tasks and maintaining balance. She had hyperreflexia, particularly on the left side. She was given BT to address underlying anemia and was put on rehabilitation therapy.

**Figure 2:**
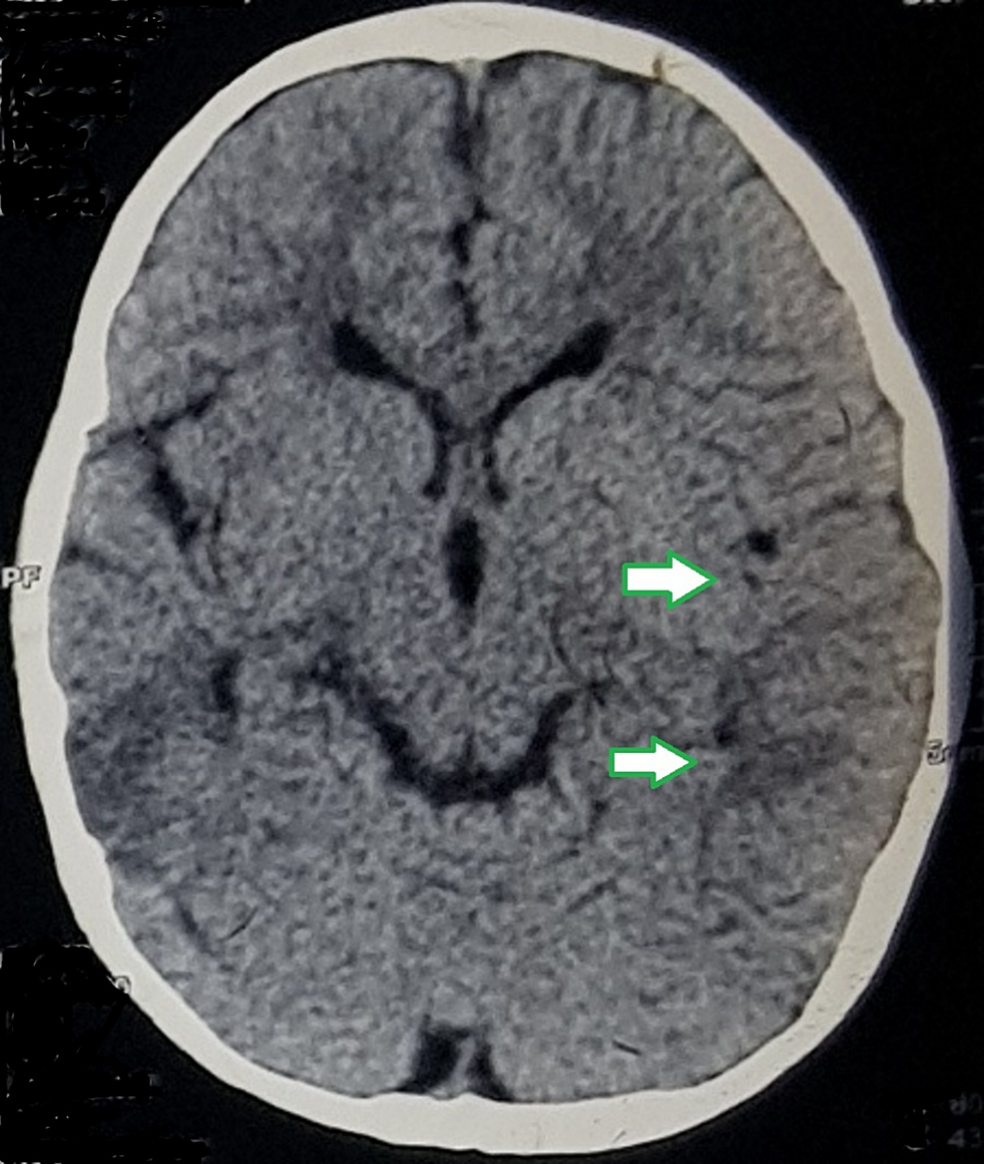
Mild ex vacuo dilatation of the ipsilateral right lateral ventricle (indicated by arrows).

**Figure 3:**
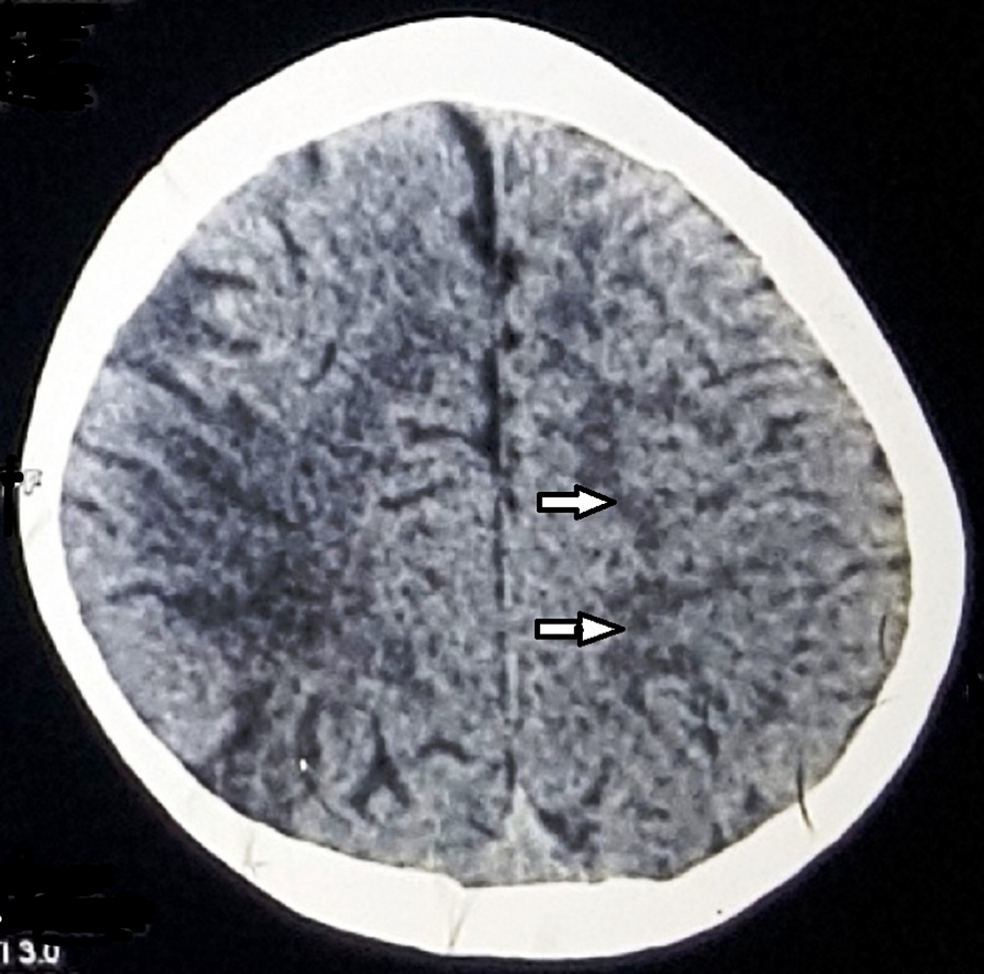
Sulcal fissures and cisterns are prominent on the right side (indicated by arrows).

The clinical findings at the time of presentation in our clinic included pallor, and the patient appeared very lethargic and mildly dehydrated with severe icterus, liver just palpable, spleen enlarged (2 cm) non-tender, reduced power in left upper and lower limbs, no koilonychia, and no hemolytic facies. Computed tomography (CT) of the brain revealed a mid-to-moderate degree of cerebral atrophy along the right temporal-parietal lobes with sulcal fissures and cisterns on the right side (Figure 2 and Figure 3).

Initial laboratory tests revealed Hb of 8.1 g (range: 12.1 - 15.1 g/dL), mean corpuscular volume (MCV) of 59 fL ( range: 80-100 fL), Mentzer’s index of 13.4, serum ferritin of 1292 ng/mL (range: 24-307 ng/mL), total bilirubin of 3.5 mg/dL (range: 0.1-1.2 mg/dL), indirect bilirubin of 2.7 mg/dL (range: 0.2-0.8 mg/dL). HPLC samples for parents were taken and the patient was started on deferasirox 1000 mg daily, Folate 5 mg, calcium, Zincovit once a day (OD), target Hb to be 8+ g and was asked to review after one week.

On the follow-up visit, patient’s symptoms aggravated and Hb dropped to 7 g. Parents' HPLC report came to be β-thalassemia carriers, i/v/o dropping Hb, and worsening symptoms. One unit saline-washed RBC transfusion was done and hydroxyurea 500 mg daily was added in addition to the previous treatment. A blood sample of the patient was drawn for DNA analysis, the next follow-up being in three weeks.

Next follow-up visit symptoms improved with Hb of 9 g/dL; the DNA of the patient came out to be homozygous for c.92+5G>C (IVS1-5G>C) confirming patient to be β-thalassemia major with an unusual clinical presentation at 12 years of age with left hemiparesis.

Currently patient is on regular third-weekly saline-washed RBC transfusions along with oral vitamin and iron chelation drugs. Five BT are done post-diagnosis with the patient’s symptoms being improved and an approximate weight gain of 4 kg in 15 weeks.

## Discussion

Middle East, Central Asia, the Indian Subcontinent, and the Far East have significant rates of cases of β-thalassemia [4]. A high prevalence of Plasmodium falciparum malaria infection is a major factor in the high case numbers in these areas. β-thalassemia significantly appears as severe anemia and a skeletal abnormality in addition to the usual clinical and radiological symptoms [5]. Chronic anemia leads to cerebral hypoxia, increasing the risk of ischemic events and stroke. Patients who are not receiving treatment or are receiving insufficient treatment have more traditional symptoms [6-7]. Patients with β-thalassemia have experienced acute neurological consequences that include cerebral ischemia, spinal cord fractures, and compression from extramedullary hematopoietic malignancies [8-9].

Ansari et al. reported right hemiparesis and a history of beta-thalassemia major in a 25-year-old female patient [10]. She had undergone frequent BT until she was nine years old and had a known instance of β-thalassemia major. The patient's mean Hb level was 9 g/dl. In our case, the patient was a 12-year-old female β-thalassemia major who had left hemiparesis at the age of five years.

The fact that thromboembolic accidents are more frequently reported with limited transfusion illustrates the beneficial function of routine transfusions. The aberrant aggregation seen with thalassemic RBCs can be eliminated by RBCs [11]. Cardioembolic and large vessel thrombosis were also documented as the cause of stroke in thalassemia, in addition to asymptomatic and symptomatic stroke due to a hypercoagulable state [12].

Iron overload is the most frequent secondary complication brought on by transfusions. This can be evaluated by looking at ferritin levels. Iron overload can result in deposition in the brain, exacerbating oxidative stress and neuronal damage. The use of chelating medicines like deferoxamine and deferasirox helps alleviate complications [13]. One can have growth retardation and an inability to mature sexually despite the use of chelating agents. The heart (dilated cardiomyopathy and pericarditis), liver (chronic hepatitis, fibrosis, and cirrhosis), and endocrine glands (diabetes mellitus, hypoparathyroidism, hypothyroidism, hypopituitarism, and low adrenal secretion) are all affected in adults with human homeostatic iron regulator protein (HFE)-associated hereditary hemochromatosis [13].

## Conclusions

Despite being a frequent ailment among South Asian populations, there are extremely few case reports on multiple circumstances. We have made an effort to report a case when the diagnosis was made but there was no follow-up. Any patient who refuses to follow-up may develop complications, so effective counseling should be provided to encourage regular follow-up and to save the patient from complications that could have been avoided with early care. Effective counseling strategies include addressing common patient concerns, emphasizing the importance of regular check-ups, and involving family members in the counseling process. Genetic counseling can help patients understand their risk of passing on the condition and assist them in making informed decisions about family planning. Additionally, the community should take an active role in helping the patients by ensuring sufficient blood supply and regular follow-up. 
